# South Africa’s Psychiatric training capacity in 2008 and in 2018. Has training capacity improved?

**DOI:** 10.4102/sajpsychiatry.29i0.1988

**Published:** 2023-03-30

**Authors:** Natalie Beath, Ugasvaree Subramaney, Zukiswa Zingela, Bonginkosi Chiliza, John A. Joska, Carla Kotzé, Suvra Ramlall, Soraya Seedat

**Affiliations:** 1 Department of Psychiatry, Faculty of Medicine and Health Sciences, Stellenbosch University, Cape Town, South Africa; 2 Department of Psychiatry, Faculty of Health Sciences, University of the Witwatersrand, Johannesburg, South Africa; 3 Faculty of Health Sciences, Nelson Mandela University, Port Elizabeth, South Africa; 4 Department of Psychiatry, School of Clinical Medicine, University of KwaZulu-Natal, Durban, South Africa; 5 Department of Psychiatry and Mental Health, University of Cape Town, Cape Town, South Africa; 6 Department of Psychiatry, Faculty of Health Sciences, University of Pretoria, Pretoria, South Africa; 7 Department of Psychiatry, Faculty of Health Sciences, University of KwaZulu-Natal, Durban, South Africa

**Keywords:** South Africa, psychiatry training, medical training, specialist training, sub-specialist training

## Abstract

**Background:**

There is a deficit of psychiatrists in South Africa, and to our knowledge, there is no situational analysis of training posts for psychiatrists in the country.

**Aim:**

To compare the number of specialists and subspecialists in training and training posts available in 2008 and 2018.

**Setting:**

South African medical schools with departments of psychiatry.

**Methods:**

A situational analysis involving data collection through a survey completed by eight heads of academic psychiatric departments followed by a comparative analysis of the two aforementioned years.

**Results:**

Data shows an 11% increase in funded and unfunded posts combined and a 9.3% increase in funded posts. The occupancy of funded posts decreased (92% in 2008 to 82% in 2018). When considering both funded and unfunded posts, only three more psychiatrists were being trained in 2018. Supernumeraries appointed in unfunded posts can be expected to return to their countries of origin. As such, a decrease in filled funded posts likely reflects a decrease in training psychiatrists destined to work in South Africa. While child and adolescent psychiatry was the only sub-speciality with accredited training posts in 2008, all sub-specialities included on the questionnaire had accredited training posts in 2018, and the number of accredited training posts in child and adolescent psychiatry doubled. That said, many of the posts were unfunded and vacant.

**Conclusion:**

While there was an increase in posts from 2008 to 2018, many posts remained unfilled. As such, not only are additional funded training posts required but also strategies to increase post-occupancy and successful completion of training.

**Contribution:**

This study is the first situational analysis of specialist and subspecialist training posts in Psychiatry in South Africa, at two time points over a 10 year period, that draws on academic heads of departments of psychiatry as respondents. The study highlights the nominal increase in funded training posts over this period, especially subspecialist training posts. The majority of Health Professions Council of South Africa (HPCSA) accredited subspecialities in Psychiatry have no funded training posts which is particularly concerning.

## Introduction

The South African Stress and Health (SASH) study, the only nationally representative study of its kind in South Africa to date, documented a 30.3% lifetime prevalence of common mental disorders in the country.^1,2^ There are no similar representative studies in South Africa other than SASH; however, international studies such as Patel et al. have shown a trend of an increasing burden of mental illness over time; in the aforementioned study there was a 41% increase in this burden over one decade.^3^ The recent COVID-19 pandemic seems to have also contributed to an increase in mental health care needs.^4^ A recent National Department of Health commissioned study recommended that in the context of decreasing the need for specialists in healthcare through a task shifting model, 3.00 psychiatrists per 100 000 population would be required to meet population needs.^5^ This is concerning as even in the context of a well-developed task-shifting model, the current ratio of 1.52–1.53 psychiatrists per 100 000 population in South Africa falls far short of population requirements.^6,7^

In South Africa, an undergraduate medical degree (generally requiring 6 years training) and completion of a 2-year internship and 1-year community service is a minimum requirement before one can apply for a specialist training post in psychiatry (an additional 4 years training).^8^ Individuals who have successfully completed their specialist psychiatry training are eligible to apply for sub-specialist training posts in Addiction Psychiatry, Old Age or Geriatric Psychiatry, Child and Adolescent Psychiatry, Neuropsychiatry, Forensic Psychiatry or Consultation-Liaison Psychiatry (an additional 2 years training).^9^

While the main examination body for specialist and subspecialist psychiatric training is the Colleges of Medicine of South Africa (CMSA) through the College of Psychiatrists, the majority of training is conducted by the nine universities registered for specialist and subspecialist training across the country. These institutions also confer the MMed degree in Psychiatry upon completion of the research requirement of the FCPsych (SA) curriculum and submission of an examined dissertation or a published peer-reviewed article. These universities include Sefako Makgatho University (SMU), Stellenbosch University (SU), University of Cape Town (UCT), University of the Free State (UFS), University of Kwazulu-Natal (UKZN), University of Limpopo (UL), University of Pretoria (UP), the University of the Witwatersrand (WITS) and Walter Sisulu University (WSU). The provincial departments of health in the nine provinces that make up South Africa are generally the main employers of registrars who are specialising or sub-specialising through funded training posts (a training post entails full-time employment with overtime requirements; trainees in funded posts are fully renumerated for their hours of employment as contracted). However, there are also training posts with registered training numbers that are not funded by the provincial department of health (unfunded), and trainees are required to source funding independently for these posts (e.g. foreign nationals from other African states who may be funded by their own government or hospital). Substantial waiting lists for funded training posts have previously been described;^10^ however, there is no recent evidence that this trend has continued.

Considering the historical and present deficits in the number of psychiatrists in the country,^6^ the authors undertook a comparison of the number and the occupancy of specialist training posts in 2008 and 2018 and compared the ratio of trainees to trainers at the respective training institutions.

## Research methods and design

The heads of department (HODs) of the nine academic psychiatry departments in South Africa were asked to provide data by means of a questionnaire. Of the nine departments, eight responded including UL, UP, UFS, WITS, SU, UKZN, WSU and UCT.

### Ethical considerations

Ethical approval was obtained from all research sites, including SU’s Health Research Ethics Committee (N18/06/063), UCT’s Faculty of Health Science Human Research Ethics Committee (155/2019), UFS (provided reciprocal review approval based on the strength of ethics clearance obtained at SU), Limpopo Department of Health and Social Science (PMREC03UL2019C), Faculty Health Sciences Research Ethics Committee, UP (537/2018), Biomedical Research Ethics Committee University of Kwazulu-Natal (RECIP401/19) and WSU Human Research Committee (072/2018). The participation of HODs was voluntary, and written informed consent was obtained from all participants. All data were stored on a password-protected device.

### Statistical analysis

All data were summarised as absolute counts and percentages using Excel.

## Results

Table 1 shows a summary of the number of Health Professions Council of South Africa (HPCSA) approved registrar training posts (funded by the Department of Health or unfunded [i.e. accredited training post numbers approved by the HPCSA but not funded by the Department of Health], vacant or filled) for the year 2008 and 2018 at the eight universities that responded to the survey. Of note, the UP and WSU did not have the relevant information available for 2008; therefore, their post numbers are included in the table but when comparing 2008–2018, UP and WSU are excluded.

**TABLE 1:** Specialist training posts.

Year	University	SA funded posts			Unfunded posts (supernumerary registrars)		
		Vacant	Filled	Total	Vacant	Filled	Total
2008	UL	0	8	8	0	0	0
	UP	-	-	-	-	-	-
	SMU	-	-	-	-	-	-
	UFS	0	12	12	0	0	0
	WITS	0	46	46	0	0	0
	SU	0	21	21	2	0	2
	UKZN	12	24	36	0	2	2
	WSU	-	-	-	-	-	-
	UCT	0	27	27	3	1	4
	Total	12	138†	150†	5	3	8
2018	UL	4	4	8	0	0	0
	UP	10	20	30	0	2	2
	SMU	-	-	-	-	-	-
	UFS	0	16	16	0	0	0
	WITS	6	48	54	0	1	1
	SU	0	21	21	2	2	4
	UKZN	18	18	36	0	3	3
	WSU	5	16	21	1	0	1
	UCT	0	29	29	2	2	4
	Total	43 (28†)	172 (136†)	215 (164†)	5 (4†)	10 (8†)	15 (12†)

University of Limpopo, UL; UP, University of Pretoria; SMU, MEDUNSA; UFS, University of the Free State; WITS, University of the Witwatersrand; SU, Stellenbosch University; UKZN, University of Kwazulu-Natal; WSU, Walter Sisulu University; UCT, University of Cape Town; UP, University of Pretoria.

† , excluding UP and WSU.

The number of available registrar posts for 2018 was 176 (164 funded and 12 unfunded), representing 18 (14 funded and 4 unfunded) additional posts and an increase of 11% from 2008 to 2018. These posts were added to the following universities, UFS (four funded), WITS (eight funded and one unfunded), UCT (two funded), SU (two unfunded) and UKZN (one unfunded). University of Pretoria (32 total, 30 funded, 2 unfunded) and WSU (22 total, 21 funded, 1 unfunded) were excluded from the aforementioned calculation as 2008 information for these institutions was not available.

An increase in vacant posts was observed when comparing 2008 and 2018. Excluding UP and WSU, in 2008: total 17 (10.8% of total available posts), 12 funded (8% of funded posts) and 5 unfunded (62% of unfunded posts) and in 2018: total 32 (18.2% of total available posts), 28 funded (17% of funded posts) and 4 unfunded (26% of unfunded posts).

The number of vacant funded posts increased at the following institutions: UL (0 in 2008, 4 in 2018), WITS (0 in 2008, 6 in 2018) and UKZN (12 in 2008, 18 in 2018), while UFS, SU and UCT had no vacant funded posts in both 2008 and 2018.

There was a small increase in the number of filled posts from 141 to 144 (138 funded and 3 unfunded in 2008 and 136 funded and 8 unfunded in 2018 (representing a 2% increase in filled posts 2008–2018 when UP and WSU were excluded from the 2018 statistics). A decrease in the number of filled funded posts was observed from 138: 2008 to 136: 2018 (representing a 1% decrease in filled funded posts from 2008 to 2018 excluding UP and WSU from the 2018 stats). As such, at the six of nine South African universities that we have data for only three more psychiatrists were trained in 2018 compared with 2008. These additional trainees were in unfunded posts, which are frequently filled by supernumeraries who are not South African and frequently return to their country of origin upon completion of training.

Table 2 summarises the available sub-specialist training posts in Child and Adolescent Psychiatry, Forensic Psychiatry, Geriatric Psychiatry and Neuropsychiatry in 2008 and 2018, funded and unfunded, vacant and filled at the eight respective universities that responded to the survey. Of note, at the time of writing, Addiction Psychiatry and Liaison Psychiatry did not have any HPCSA-approved training posts and, as such, no funded or unfunded posts for these sub-specialities existed. They were offered at some institutions as an MPhil degree programme; however, as this was not asked in the survey, the number of trainees registered for MPhil degree programmes was not captured.

**TABLE 2:** Sub-specialist training posts.

Year Sub-specialty	University	Funded vacant	Unfunded				
			Filled	Total	Vacant	Filled	Total
2008	UL	0	0	0	0	0	0
Child Psychiatry	UP	-	-	-	-	-	-
	UFS	0	0	0	0	0	0
	WITS	0	4	4	0	0	0
	SU	0	1	1	1	0	1
	UKZN	0	0	0	0	0	0
	WSU	0	0	0	0	0	0
	UCT	1	1	2	0	1	1
Total		1	6	7	1	1	2
2018	UL	0	0	0	0	0	0
Child Psychiatry	UP	1	0	1	0	0	0
	UFS	1	0	1	0	0	0
	WITS	4	2	6	0	0	0
	SU	0	1	1	3	0	3
	UKZN	0	0	0	0	0	0
	WSU	0	0	0	0	0	0
	UCT	1	0	1	5	0	5
Total		7	3	10	8	0	8
Forensic Psychiatry	UL	0	0	0	0	0	0
	UP	6	0	6	0	0	0
	UFS	0	0	0	0	0	0
	WITS	5	3	8	0	0	0
	SU	0	0	0	0	0	0
	UKZN	0	0	0	0	0	0
	WSU	0	0	0	0	2	2
	UCT	0	0	0	1	1	2
Total		11	3	14	1	3	4
Geriatric Psychiatry	UL	0	0	0	0	0	0
	UP	0	0	0	0	0	0
	UFS	0	0	0	0	0	0
	WITS	0	0	0	0	0	0
	SU	0	0	0	1	1	2
	UKZN	0	0	0	0	0	0
	WSU	0	0	0	0	0	0
	UCT	0	0	0	0	0	0
Total		0	0	0	1	1	2
Neuropsychiatry	UL	0	0	0	0	0	0
	UP	0	0	0	0	0	0
	UFS	0	0	0	0	0	0
	WITS	4	1	5	0	0	0
	SU	0	0	0	2	0	2
	UKZN	0	0	0	0	0	0
	WSU	0	0	0	0	0	0
	UCT	0	1	1	0	1	1
Total		4	2	6	2	1	3

Note: Geriatric Psychiatry, Neuropsychiatry and Forensic Psychiatry had not been approved as of yet in 2008 and as such there were no funded posts at any institution. Child Psychiatry was the only accredited subspecialty

UL; University of Limpopo; UP, University of Pretoria; UFS, University of the Free State; WITS, University of the Witwatersrand; SU, Stellenbosch University; UKZN, University of Kwazulu-Natal; WSU, Walter Sisulu University; UCT, University of Cape Town.

The only subspeciality in psychiatry that had training posts in 2008 was Child and Adolescent Psychiatry with nine training posts (seven funded and two unfunded; no 2008 data were available for UP). Child and Adolescent Psychiatry had only two vacant posts in 2008 (22% of total available posts) (one funded and one unfunded). By 2018, the number of Child and Adolescent Psychiatry training posts had doubled to 18 training posts in total (10 funded and 8 unfunded). Of these, 15 posts were vacant (83% of total available posts) (seven funded and eight unfunded). Despite the increase in posts in Child and Adolescent Psychiatry, fewer child and adolescent psychiatrists were being trained in 2018 compared with 2008 (seven in 2008 and three in 2018).

In 2018, Forensic Psychiatry had 18 training posts (14 funded and 4 unfunded), with 12 vacant (66.7% of total available posts) (11 funded and 1 unfunded). Geriatric Psychiatry had two training posts (two unfunded) in 2018, one filled and one vacant. Neuropsychiatry had nine training posts (six funded, three unfunded), six were vacant (66.7% of total available posts) (four funded, two unfunded).

Table 3 summarises the number of consultants and registrars at each of the institutions at the time of the survey (2019–2020). The ratio of consultants to registrars at each institution is presented. Of note, all the universities have more registrars than consultants, except for UCT and UP with both having a 1:1 ratio, WITS (one consultant: 0.97 registrars) and UKZN (one consultant: 0.95 registrars). That said, considering the vacant registrar posts at UL, UP, WITS, UKZN and WSU, registrars would outnumber consultants at all the universities except for UCT should those posts be filled. However, should all the registrar posts be filled, the ratio of registrar to the consultant would still fall short of the HPCSA stipulated maximum of four registrars to one consultant, indicating a capacity for increased registrar posts.

**TABLE 3:** Number and ratio of consultants and registrars at institutions.

University	Facility	Number of registrars	Number of consultants	Consultant: Registrar ratio
UL	Thabamoopo	4	3	1:1.33
	Mankweng	2	2	1:1
Total		6	5	1:1.2
UP	Weskoppies	16	16	1:1
	Steve Biko	4	3	1:1.33
	Witbank	0	0	-
	Tembisa	0	1	-
Total		20	20	1:1
UFS	Pelonomi	2	2	1:1
	FSPC	14	7	1:2
Total		16	9	1:1.78
WITS	CMJAH	6	7	1:0.86
	HJH	4	3	1:1.33
	CHBAH	14	14	1:1
	SBAH	12	19	1:0.63
	Tara hospital	9	9	1:1
	Community	12	7	1:1.71
	RMMCH	1	1	1:1
Total		58	60	1:0.97
SU	TBH	8	6	1:0.75
	SLH	9	9	1:1
	LGH	4	4	1:1
	District	0	1	1:0
Total		21	20	1:1.05
UKZN	King Edward	3	3	1:1
	Addington	4	3	1:1.33
	King DiniZulu	6	4	1:1.5
	Town Hill	6	6	1:1
	Fort Napier	1	3	1:0.33
	Ngwelezana	1	3	1:0.33
Total		21	22	1:0.95
WSU	Nelson Mandela CH	4	5	1:0.8
	Dora Nginza Hospital	7	2	1:3.5
	Komani Hospital	0	1	-
	Elizabeth Donkin Hospital	3	2	1:1.5
	Fort England Hospital	4	5	1:0.8
Total		18	15	1:1.2
UCT	VBH	11	11	1:1
	LGH	6	6	1:1
	RXH	3	3	1:1
	GSH	9	6	1:1.5
	Alexandra Hospital	1	1	1:1
	SLH	1	1	1:1
	Community	-	3	-
Total		31	31	1:1

UL, University of Limpopo; UP University of Pretoria; UFS, University of the Free state; SU, Stellenbosch University; UKZN, University of Kwazulu-Natal; WSU, Walter Sisulu University; UCT, University of Cape Town; WITS, University of the Witwatersrand; FSPC, Free State Psychiatric Complex; CMJAH, Charlotte Maxeke Johannesburg Academic Hospital; HJH, Helen Joseph Hospital; CHBAH, The Chris Hani Baragwanath Hospital; SBAH, Steve Biko Academic Hospital; RMMCH, Rahima Moosa Mother and Child Hospital; TBH, Tygerberg Hospital; SLH, Stikland Hospital; LGH, Lentegeur Hospital; VBH, Valkenburg Hospital; RXH, Red Cross War Memorial Children’s Hospital; GSH, Groote Schuur Hospital.

In the final section of the survey, HODs were asked if they believed the number of training posts at their respective institutions was adequate to meet population needs. All commented that there were shortages, particularly highlighting the lack of funded sub-specialist training posts.

## Discussion

While long waiting lists for specialist psychiatric training posts were previously mentioned,^10^ this is to the authors knowledge the first situational analysis of the number of specialist and sub-specialist psychiatry training posts in South Africa. Based on comparable data over one decade, there appears to have been a 9.3% increase in funded specialist psychiatry training posts from 2008 to 2018 (150 in 2008 to 164 in 2018). During the same period, the percentage occupancy of funded posts did not increase (138/150=92% in 2008 to 136/164=82% in 2018), with 82% of funded posts filled in 2018 compared with 92% in 2008, indicating a decrease in trainee psychiatrists in South Africa. With a total number of filled posts in 2018 (excluding the university that did not respond to the survey) being 182 (172 funded, 10 unfunded) and making the assumption that these posts are equally distributed over the 4 years of training, this represents about 45.5 training specialists per year of training. According to the CMSA stats office, the average pass rate between 2012 and 2021 (inclusive) was 54.68% over 10 years of examinations. This equates to 24.88 final-year candidates successfully completing their training each year. In comparison, the average pass rate 2012–2021 for the other seven major colleges is as follows:

- Fellowship of the College of Anaesthetists of South Africa (FCA[SA]) 58%
- Fellowship of the College of Neurosurgeons of South Africa (FC Neurosurg [SA]) 52%
- Fellowship of the College of Obstetricians and Gynaecologists of South Africa (FCOG[SA]) 65%
- Fellowship of the College of Orthopaedic Surgeons of South Africa (FC Orth [SA]) 64.5%
- Fellowship of the College of Paediatricians of South Africa (FC Paed [SA]) 65%
- Fellowship of the College of Physicians of South Africa (FCP([SA]) 50.7%
- Fellowship of the College of Surgeons of South Africa (FCS[SA]) 67%.

The pass rates over this period are concerning considering: (1) the need to increase the psychiatrist-to-population ratio in the context of a growing population (1.3% per year)^11^ and (2) qualified psychiatrists leaving practice in South Africa either because of retirement (n: 33; 4.5%, at retirement age)^1^ or emigration.^12^ Many of the unfunded posts are occupied by foreign trainees who are likely to leave South Africa after their training is completed and therefore will have no sustained impact on the psychiatrist-to-population ratio in the country.

If it is assumed that the South African population in 2019 was 58 558 270^13^ and three psychiatrists are required per 100 000 population, this would equate to a total of 1756.75 psychiatrists. In 2019, the active population of psychiatrists is said to have been 793,^6^ yielding a shortfall of 96 375 psychiatrists. If it is assumed that all the funded training posts are filled (215), all final year trainees (215/4=53.75) pass their training successfully in the 4 years of training time, no psychiatrists retire or emigrate and the South African population does not grow, it would take 17.9 years to make up the shortfall in psychiatrists in South Africa with the current post allocations.

While there were no subspecialist training posts except for Child and Adolescent Psychiatry in 2008, all the subspecialities that were included in the questionnaire had HPCSA-accredited training posts in 2018, with the number of training posts in Child and Adolescent Psychiatry doubling over a 10-year period. Despite this increase, there were fewer Child and Adolescent psychiatrists being trained in 2018 compared with 2008. Sadly, many of the training posts are unfunded – geriatrics, for example, has no funded training posts yet with an ageing South African population,^14^ it can be expected that the mental healthcare needs of this population will also increase. The majority of available subspecialist training posts were vacant in 2018. To date, there are very few HPCSA-approved training post numbers for Addiction Psychiatry, yet South Africa has very high rates of substance abuse with marked psychosocial impact,^15,16,17,18^ while the treatment of substance use disorders has been shown to be cost effective.^19^

There are a variety of factors that may be contributing to general medical practitioners and specialist psychiatrists not pursuing specialist and subspecialist training. Some that have been mentioned in the past include HPCSA’s decisions on which academic units should be allocated specialist and subspecialist training posts and which subspecialities get accredited, as well as limiting the total number of subspecialist training posts per institution to two per subspecialist (for each subspecialist employed by an academic department the possible number of subspecialist training posts for that particular subspeciality that the academic department can apply for is capped at two) and the National Department of Health and the Department of Higher Education and Training being the only bodies that can authorise training of specialists and sub-specialists with training only possible through the public healthcare sector as a 4-year (specialist) and 2-year full-time or 4-year part-time course (sub-specialist).^20^ As such, general practitioners and psychiatrists working in the private sector would have to leave their private practices and make economic sacrifices to pursue specialist and sub-specialist training, respectively. A possible solution that has been suggested is public–private partnerships, with trainees working in both the private and public sectors and receiving subspeciality exposure in both settings but also jointly drawing on private and public entities for potential funding for such training. Alternatively, making more widely available the 4-year part-time subspecialist training option, allowing trainees to continue their private practice. Another obstacle to specialising or sub-specialising may be the need to relocate to cities with universities that offer specialist and sub-specialist training. Distanced learning may help to alleviate this problem with the possibility of an adapted curriculum where decentralised facilities with remote psychiatrist supervisors can be accredited, allowing trainees to work in their home practice environment for extended periods and spend shorter, concentrated periods at an academic institution. Not having to relocate has the additional benefit of possibly distributing specialists and sub-specialists across the country in a more equitable manner as specialists and sub-specialists are currently concentrated in geographic pockets of South Africa,^6^ likely close to where they originally specialised. Of note, the majority of registrars training in psychiatry indicated in a survey conducted in 2019 that they intended to remain in the province they were located in during their training once their training was completed.^18^ Considering the aforementioned, merely adding training posts to university hospitals that do not have vacant posts would not solve the problem of inequitable distribution of psychiatrists across the country. The reasons why certain universities have vacant posts and others not are not clear but may include (1) location universities located in cities with qualities that trainees may find desirable for themselves or to raise a family in. Thus, being located near large cities where many general practitioners or specialist psychiatrists already reside and not needing to relocate to pursue training. Related to this may be familiarity with a training institution (e.g. choosing the institution where undergraduate training was undertaken). (2) Qualities of the training institution itself – a survey among training psychiatrists in 2019 indicated significant variability in trainees’ perception of the quality of their training and resources at their training facilities.^18^

Differences across institutions in the numbers of filled and vacant posts in 2008 and 2018 are difficult to explain considering that the data presented here reflect two time points rather than longitudinal year-on-year estimates. Drawing comparisons and providing reasons for the discrepancies and the potential impact would, therefore, be purely speculative. Filling of registrar posts is a dynamic annual process with different institutions in the country filling registrar posts at different times in the calendar year. That said, the increase in training post numbers nationally (which in the absence of an increase in provincially funded registrar posts across the country, over time) represents a strategic effort on the part of university departments of psychiatry to increase capacity on the service training platform in light of the increase in mental health service demands.

Shortcomings of the study include that not all nine universities that provide specialist and sub-specialist psychiatry training in South Africa responded to the survey of this study and of the universities that did respond some did not have comparable data for both 2008 and 2018. As such, the actual increase in the number of specialist and sub-specialist training posts from 2008 to 2018 was difficult to determine. In addition, SMU prior to 2015 was known as MEDUNSA campus and was part of the Limpopo University. In 2015, MEDUNSA campus split from the Limpopo University and became SMU. Thus, any posts added in 2018 through the establishment of SMU in 2015 must be viewed with this background in mind. That said, the eight universities that did respond to the questionnaire represent the largest universities in the country that offer registrar training in Psychiatry. The data represent a cross-sectional analysis covering two calendar years approximately 10 years apart and may not be representative of sustained trends between 2008 and 2018, and lastly, HODs may have also completed the questionnaire without consulting their human resource department or personnel records, resulting in inaccurate data.

## Conclusion

While long waiting lists for specialist psychiatry training posts were previously described, this situation analysis shows multiple vacant specialist posts at training institutions in the country. Despite an increase in the number of specialist psychiatry training posts from 2008 to 2018, South Africa is unlikely to reach the recommended number of three psychiatrists per 100 000 population with the current funded post allocations in the next two decades if retirement, migration and population growth are considered, even if all vacant posts were filled, and all trainees have successfully completed their training within the stipulated time frame. Not only are additional funded training posts required but also strategies to increase post occupancy, retention and successful (and timeous) completion of training.
